# Metagenome reveals potential microbial degradation of hydrocarbon coupled with sulfate reduction in an oil-immersed chimney from Guaymas Basin

**DOI:** 10.3389/fmicb.2013.00148

**Published:** 2013-06-14

**Authors:** Ying He, Xiang Xiao, Fengping Wang

**Affiliations:** ^1^State Key Laboratory of Microbial Metabolism, School of Life Sciences and BiotechnologyShanghai, China; ^2^State Key Laboratory of Ocean Engineering, Shanghai Jiao Tong UniversityShanghai, China

**Keywords:** metagenome, deep sea, hydrothermal vent, chimney, Guaymas, biodegradation, hydrocarbon, sulfate reduction

## Abstract

Deep-sea hydrothermal vent chimneys contain a high diversity of microorganisms, yet the metabolic activity and the ecological functions of the microbial communities remain largely unexplored. In this study, a metagenomic approach was applied to characterize the metabolic potential in a Guaymas hydrothermal vent chimney and to conduct comparative genomic analysis among a variety of environments with sequenced metagenomes. Complete clustering of functional gene categories with a comparative metagenomic approach showed that this Guaymas chimney metagenome was clustered most closely with a chimney metagenome from Juan de Fuca. All chimney samples were enriched with genes involved in recombination and repair, chemotaxis and flagellar assembly, highlighting their roles in coping with the fluctuating extreme deep-sea environments. A high proportion of transposases was observed in all the metagenomes from deep-sea chimneys, supporting the previous hypothesis that horizontal gene transfer may be common in the deep-sea vent chimney biosphere. In the Guaymas chimney metagenome, thermophilic sulfate reducing microorganisms including bacteria and archaea were found predominant, and genes coding for the degradation of refractory organic compounds such as cellulose, lipid, pullullan, as well as a few hydrocarbons including toluene, ethylbenzene and o-xylene were identified. Therefore, this oil-immersed chimney supported a thermophilic microbial community capable of oxidizing a range of hydrocarbons that served as electron donors for sulphate reduction under anaerobic conditions.

## Introduction

Deep-sea hydrothermal vents characterized by steep physico-chemical gradients harbor a wide range of microorganisms in different ecological niches, including the high-temperature chimney matrix (Reysenbach and Shock, 2002). Deep-sea hydrothermal vent chimneys are the product of hydrothermal circulation and alteration of seawater entrained through geothermally heated subseafloor basalt, and subsequent precipitation of mental sulfides when hot vent fluids emerge into cold sea water (Von Damm, 1990). The geochemical disequilibria within and surrounding chimneys provide rich energy sources for microorganisms, as various reduced chemicals (such as sulfur, methane, and H_2_) are utilized as potential electron donors (Jannasch and Mottl, 1985; Distel et al., 1988; Lam et al., 2004; Petersen et al., 2011). The structures of these microbial communities are shaped primarily by variation of hydrothermal fluid composition, in particular H_2_ concentrations (Flores et al., 2011). Thanks to advances in sequencing technologies, molecular microbial diversity studies of deep-sea hydrothermal environments have made significant progress in understanding the geographic distributions of these microbial communities (Teske et al., 2002; Huber et al., 2007, 2010; Brazelton et al., 2010; Dick and Tebo, 2010; Roussel et al., 2011).

Despite the increased knowledge of the microbial diversity of deep-sea hydrothermal vents, much less was known about the metabolic potential and ecological functions of these communities, especially when considering that less than 1% of environmental microorganisms could be cultured under laboratory conditions (Amann et al., 1995). Metagenomic-based methods have provided unique opportunities to explore the features of microbial communities from diverse deep-sea hydrothermal vent environments. So far, metagenomes from two deep-sea hydrothermal vent chimneys have been published, from a carbonate white chimney at Lost City with relatively low temperature and high pH (<90°C, pH 9–11) (Brazelton and Baross, 2009), and from a chimney sample collected from Juan de Fuca (Xie et al., 2011) characterized by high temperature and low pH fluids (>300°C, pH 2–3). Both metagenomes were found enriched in transposases, implying that horizontal gene transfer may be a common feature of hydrothermal vent chimney biosphere. Comparative metagenomic studies with more chimney samples from different deep-sea hydrothermal vents should be performed to reveal common features of the chimney-originated microbial communities.

The Guaymas Basin (Gulf of California) is a unique hydrothermal vent site, where emitted high-temperature fluids are influenced by the presence of a thick layer (100–500 m) of sediments (with 2–4% organic matter, OM). These sediments were formed by precipitation from the highly productive surface waters and terrigenous input (Von Damm et al., 1985). The venting fluids are characterized by an increase of pH (around 6.0), and a decrease in the highest temperature of fluids emitted on the seafloor (270–325°C) (Von Damm et al., 1985). Therefore, the chimney sample from the Guaymas site would be an ideal sample for comparative metagenomic analysis together with the two published ones. In addition, Guaymas Basin is unique due to the fact that under high-temperature conditions, the OM in its rapidly accumulating sediments is pyrolized to petroleum-like hydrocarbon products, such as aliphatic and aromatic hydrocarbons, short-chain fatty acids, ammonia, and methane (Bazylinski et al., 1988; Welhan, 1988; Martens, 1990; Kawka and Simoneit, 1994). The hydrothermally active sediments of the Guaymas Basin were reported with intensive methane oxidizing and sulfate reducing activities (Jørgensen et al., 1990, 1992; Elsgaard et al., 1994; Weber and Jørgensen, 2002; Kallmeyer and Boetius, 2004). Various hydrocarbon degrading microorganisms which are using sulfate as the electron acceptor have been isolated from Guaymas Basin sites and similar marine habitats (Rüter et al., 1994; Galushko et al., 1999; Musat and Widdel, 2008; Kleindienst et al., 2012). Nevertheless, the metabolic potential, in particular for hydrocarbon degradation, of the whole microbial community from Guaymas vent chimneys has never been investigated. Therefore, comprehensive studies on biodegradation, in particular under anaerobic conditions, still remain to be conducted to characterize the structure and metabolic potential of microbial ecosystems with capability for hydrocarbon biodegradation.

In this study, the metagenome of an oil-immersed chimney in Guaymas Basin was analyzed to demonstrate the metabolic potential and ecological functions of the inhabited microbial community. Additionally, questions related to anaerobic biodegradation of hydrocarbons are addressed: do the microorganisms from this chimney community have anaerobic hydrocarbon degradation activities? If so, what kinds of hydrocarbons could be potentially degraded? Which groups of microorganisms are responsible for carrying out the degradations? Which processes are coupled/closely related to the degradation for electron transfer? Are there any features shared among different chimney-originated samples?

## Materials and methods

### DNA extraction and sequencing

**Figure A1:**
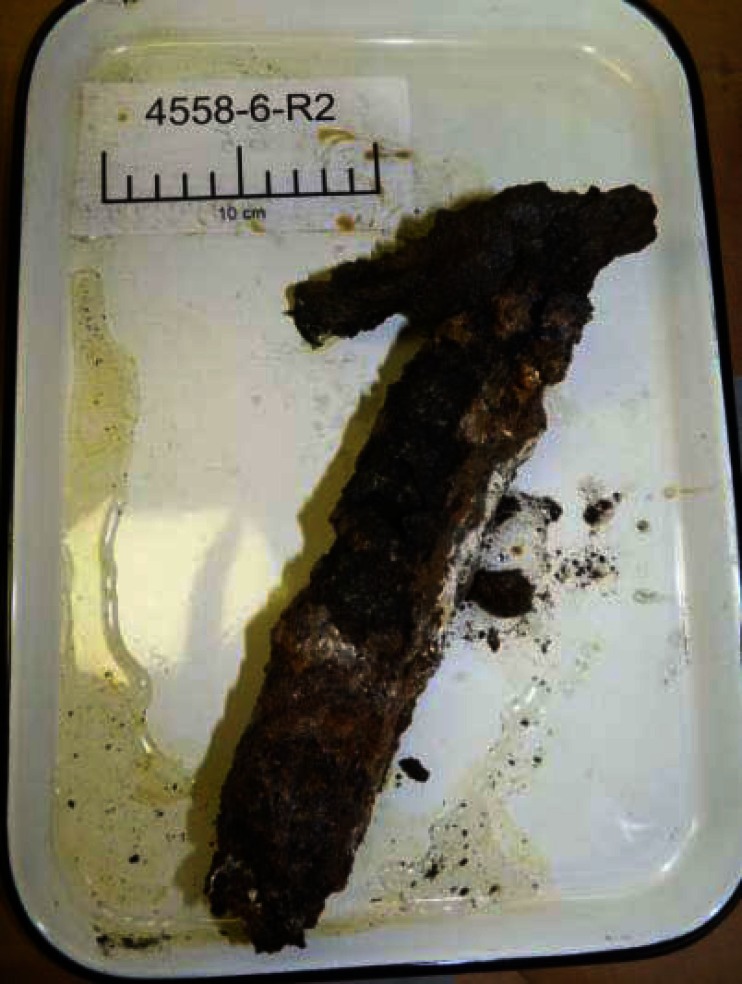
**The photo of a black-smoker chimney sample (4558-6), collected in Guaymas Basin (27°0.9′N, 111°24.6′W, depth = 2013 m)**.

The sample 4558-6 under investigation represented the outer layer of a black-smoker chimney with preliminary venting fluid temperature 190°C (measured above the chimney prior to sampling), and was collected in Guaymas Basin (27°0.9′N, 111°24.6′W, depth = 2013 m) by the HUV Alvin (supported by the R/V Atlantis) in November, 2009. The chimney (Figure A1) was kept at −20°C immediately after sample collection, and stored with dry ice during transportation and stored at −80°C in laboratory until further analyses. The genomic DNA was extracted from outer sections of all collected samples where highest DNA quantity was found. Isolation of DNA was conducted as described in a previous study (Wang et al., 2009). Metagenome pyrosequencing was performed according to company protocol on the 454 Life Sciences GS FLX system with a practical limit of 400 bp. All the sequences were deposited in the MG-RAST server.

### Metagenomic analysis and assembly

Low quality sequencing reads were trimmed in Geneious 6.04 (Biomatters Ltd.) with default parameters. Technical replicates (Gomez-Alvarez et al., 2009) were removed with cd-hit (at 96% sequence identity) (Niu et al., 2010). Shorter reads (<100 bp) were then excluded, and the remaining reads were assembled with Velvet (Zerbino and Birney, 2008). This metagenome from Guaymas Basin chimney was uploaded (MG-RAST ID: 4510962.3) and analyzed using MG-RAST (Meyer et al., 2008).

### Metagenomic sequence analysis

Coding regions within the metagenome were predicted using FragGeneScan (Rho et al., 2010), and the predicted sequence features were then annotated (e-value <1e-5) against M5NR protein database. 16S rRNA genes were predicted with HMM and BLASTN (e-value <1e-5) in webMGA (Wu et al., 2011), respectively. To analyze the taxonomic contents, all predicted gene features were subject to blastx (Altschul et al., 1997) searches against NCBI non-redundant (NR) database (e-value <1e-5, word size = 3, multi hit window size = 40 and low complexity filter on) and visualized in MEGAN (Huson et al., 2007). Each predicted sequence feature in the metagenome was assigned to a certain taxon when at least 75% of the BLAST hits of this query were from that specific taxon. Sequences with matches to the eggNOG (Powell et al., 2012), COG (Tatusov et al., 2003) and KEGG (Ogata et al., 1999) database were retrieved to build functional categories and reconstruct metabolic pathways.

### Annotation of sequences with degradation/metabolic activities

Sequences that were annotated as enzymes in the degradation of cellulose and fatty acids were extracted and subject to manual examination. Annotated KEGG pathways in this metagenome were visualized in MEGAN to demonstrate the metabolic potential in the microbial community. Enzymes involved in the degradation of a few organic chemicals (such as benzene, toluene, ethylbenzene, and xylenes) were collected from the Biocatalysis/Biodegradation Database (BBD) of the University of Minnesota (Gao et al., 2010), an online web service that listed known degradation pathways for hundreds of chemicals/contaminants. Within this chimney metagenome, a sequence-similarity based search against BBD was conducted to identify candidate genes that were involved in the biodegradation of certain hydrocarbons. Sequences of previously reported anaerobic alkane degradation genes (Callaghan et al., 2010), benzylsuccinate synthase (*bss*) and alkylsuccinate synthase (*ass*), were retrieved from GenBank (accession no.: DQ826035, DQ826036, AJ001848, AB066263, AY032676, and AF113168) and searched against our Guaymas metagenome.

### Clustering analysis of functional categories

Clustering of functional categories (with KEGG annotation, e-value < 1e-5, min. identity of 30% and min. align. length of 15 a.a.) was conducted in MG-RAST (using ward with canberra distance metric based on normalized values) among metagenomes as following: Guaymas Basin chimney 4558-6 (MG-RAST ID: 4510962.3), Juan de Fuca chimney (MG-RAST ID: 4510965.3) (Xie et al., 2011), Lost City hydrothermal vent field (MG-RAST ID: 4461585.3) (Brazelton and Baross, 2009), two biofilm samples from acid mine drainage (UBA and 5way, with MG-RAST ID 4441137.3 and 4441138.3, respectively) (Tyson et al., 2004), a gutless worm (MG-RAST ID: 4441115.3) (Woyke et al., 2006), the North Pacific Subtropical Gyre microbial communities (the HOT project with depth of 10, 70, 130, 200, 500, 770 and 4000 m, and with MG-RAST ID: 4441051.3, 4441057.4, 4441055.3, 4441041.3, 4441057.3, 4441062.3, and 4441056.3, respectively) (Delong et al., 2006) and a deeply buried sediments from Peru Margin (for sample original, am1mbsf, am16mbsf, am32mbsf and am50mbsf, and with MG-RAST ID: 4440960.3, 4440961.3, 4440973.3, 4459940.3, and 4459941.3, respectively) (Biddle et al., 2008). All metagenomes were stored in MG-RAST database.

## Results

### Sequencing summary and coverage

**Figure 1:**
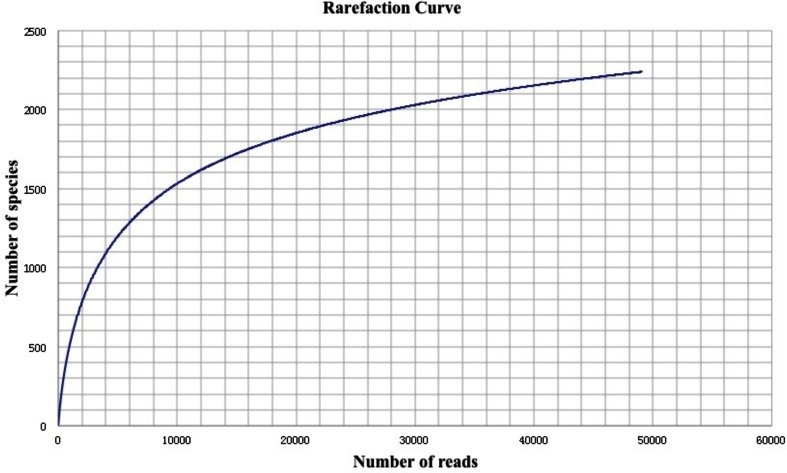
**Rarefaction plot of the total number of distinct taxon annotations (MG-RAST) as a function of the number of sequences from the assembly**.

Initially, as shown in Table 1, a total amount of 512,830 reads and 196,377,880 bp of sequence data were generated by 454 pyrosequencing. After removing low-quality reads and technical duplicates, the remaining 504,915 reads (with an average length 383 bp) were assembled into 49,055 contigs (totaling 26,241,624 bp, with an average length of 543 bp). 187,308 singletons with an average length of 367 bp could not be assembled. From this assembly, 52,366 gene features were predicted, 37,372 of which (71.4%) were with known annotations. A rarefaction analysis of the final assembly was conducted based on the taxonomic information retrieved from annotation results in MG-RAST (Figure 1), which indicated that a reasonable number of individual genomes were sampled and covered in the metagenome.

**Table 1:** **Summary of sequences from the chimney sample 4558-6**.

**Properties**	**Value**
Clean reads	504,915
Base pairs	193,336,182
GC content	42%
Average read length	383
Singletons	187,308
Contigs	49,055
Average contig length	543
Best hit gene features with NR database	37372

### Taxonomic diversity based on 16s rRNA gene prediction

70 and 90 16S rRNA sequences were predicted from webMGA (Wu et al., 2011), with the use of HMM and BLASTN, respectively (Table 2). In both of the results, around 3/4 (72.9 and 76.7%, respectively) of all identified 16S rRNA sequences were from bacteria. Deltaproteobacteria and Euryarchaeota had dominated the bacteria and archaea, respectively.

**Table 2:** **Taxonomic diversity based on 16S rRNA gene prediction from the metagenome**.

**Methods**	**Domain [total no. of sequences (%)]**	**Diversity (% in domain)**
HMM	Bacteria (51, 72.9)	Deltaproteobacteria (25.4)
		Gammaproteobacteria (21.6)
		Epsilonproteobacteria (13.7)
		Deferribacteres (7.8)
	Archaea (19, 27.1)	Euryarchaeota (63.2)
		Crenarchaeota/Thermoprotei (36.8)
BLASTN	Bacteria (69, 76.7)	Deltaproteobacteria (23.4)
		Gammaproteobacteria (18.8)
		Epsilonproteobacteria (12.5)
		Acidobacteria (9.4)
		Deferribacteres (7.8)
	Archaea (21, 23.3)	Euryarchaeota (52.4)
		Crenarchaeota/Thermoprotei (47.6)

### Phylogeny from 454 pyrosequencing data

**Figure 2:**
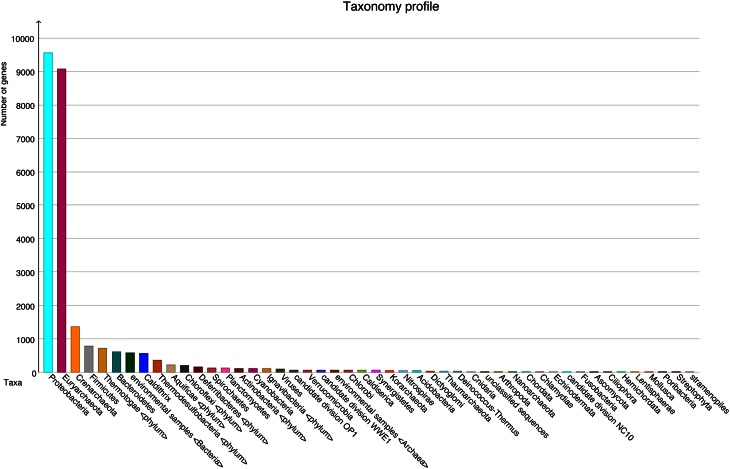
**Taxonomic information at the phylum level with number of sequences in each phylum, based on BLASTX (1e^−5^) results**.

A total number of 37,372 predicted gene features were utilized to extract taxonomic information by BLASTing against the NCBI NR database. In total, 21,792 (58.3%) and 11,249 (30.1%) hits were assigned to bacteria and archaea (rules for taxonomic assignment were stated in “Materials and Methods”), respectively (Table A1). At the phylum level, Proteobacteria (9558, 25.6%), Euryarchaeota (9078, 24.3%), Crenarchaeota (1354, 3.6%), Firmicutes (786, 2.1%), Thermotogae (725, 1.9%), and Bacteroidetes (607, 1.6%) had the top matches (Figure 2). Taxa with most hits assigned were listed in Table 3. Notably, the majority of sequences in this chimney metagenome originated from the families *Archaeoglobaceae, Thermococcaceae*, and *Desulfobacteraceae*, and a high proportion (>21.2%) of sequences potentially originated from sulfate reducing prokaryotes (SRP). Moreover, thermophilic sulfate reducing microorganisms, including bacteria (*Thermodesulfobacteriaceae*) and archaea (*Archaeoglobaceae*) were found predominant, highlighting their roles in reducing sulfates and/or sulfites to sulfides during energy metabolism under anaerobic conditions.

**Table 3:** **Top families with the most hits assigned**.

**Family**	**No. of hits assigned (%)**	**Phylum**
*Archaeoglobaceae*	4777 (12.78)	Euryarchaeota
*Thermococcaceae*	2365 (6.33)	Euryarchaeota
*Desulfobacteraceae*	2117 (5.66)	(Delta) Proteobacteria
*Thermotogaceae*	723 (1.93)	Proteobacteria
*Desulfurococcaceae*	672 (1.80)	Crenarchaeota
*Helicobacteraceae*	375 (1.00)	(Epsilon) Proteobacteria
*Thermodesulfobacteriaceae*	355 (0.95)	Proteobacteria
*Colwelliaceae*	344 (0.92)	(Gamma) Proteobacteria
*Thiotrichaceae*	214 (0.57)	(Gamma) Proteobacteria
*Desulfovibrionaceae*	208 (0.56)	(Delta) Proteobacteria
Sulfate reducing prokaryotes	7921^*^ (21.2)	–

The exact number of blast hits was listed for each family, with the percentages (out of total hits) listed in the parentheses.

* The number of hits for SRP was generated by summing all the hits from Archaeoglobaceae, Desulfobacteraceae, Desulfurococcaceae, Desulfovibrionaceae, and Thermodesulfobacteriaceae.

### Functional category hits distribution

Major KEGG function categories were listed (Table 4) and ordered by the number of unique hits assigned to each category. Similar to a previous study on the chimney from hydrothermal vents (Xie et al., 2011), genes involved in Recombination and Repair were among the abundant categories (Table 4). In addition, transposaes were found to be highly enriched in this sample (Table A3), and genes participated in Chemotaxis and Flagellar Assembly was all identified with high abundance in this metagenome.

**Table 4:** **List of major KEGG families**.

**KEGG (level 2) annotation**	**Avg. % ident**	**Avg. aligned len.**	**No. of hits**
Amino acid metabolism	69.68	80.99	1756
Carbohydrate metabolism	69.96	75.17	1365
Translation	71.01	66.33	1301
Membrane transport	68.01	70.23	836
Metabolism of cofactors and vitamins	68.73	71.72	723
Energy metabolism	72.02	73.84	689
Nucleotide metabolism	68.99	68.01	550
Replication and repair	67.7	71.41	515
Folding, sorting and degradation	71.9	71.82	386
Signal transduction	67.86	62.85	375
Cell motility	70.33	57.04	258
Glycan biosynthesis and metabolism	67	65.77	240
Transcription	73.36	75.02	213
Cell growth and death	68.78	64.14	209
Lipid metabolism	70.28	64.61	184

Average sequence identity (Avg % ident) and aligned sequence length (Avg aligned len) were presented. No. of Hits represented the number of unique hits within each functional protein category. Uncharacterized proteins were excluded.

### Degradation of refractory OM and petroleum hydrocarbons

Sequences coding for the complete degradation pathway of cellulose and fatty acids, as well as key enzymes involved in breakdown of lipid and pullulan were all identified in this metagenome (Table A2). Many of these identified enzymes were with thermorphilic-origin best hits (as marked with asterisk in Table A2). Peptidases were found to be of low abundance in this metagenome (data not shown). For anaerobic hydrocarbon degradation, sequences coding for benzylsuccinate synthase (*bss*) and alkylsuccinate synthase (*ass*), key enzymes in the fumarate addition pathway for the anaerobic oxidation of hydrocarbons (Callaghan et al., 2010), were identified (Table 5). In particular, gene candidates involved in BTEX (benzene, toluene, ethylbenzene, and xylenes) degradation were identified by searching against the BBD of the University of Minnesota (Gao et al., 2010) (Table 5). Additionally, to show the metabolic potential of this Guaymas sample in the degradation and remediation of organic contaminants (He et al., 2010), genes involved in the degradation of aromatic carboxylic acid (benzoate, phenylpropionate and phthalate), chlorinated aromatics (2- and 4-chlorobenzoate, 2,4,5-trichlorophenoxyacetic acid), heterocyclic aromatics (carbazole and dibenzothiophene), nitroaromatics (nitrobenzene and nitrophenol) as well as a few of other hydrocarbons (cyclohexane and tetrahydrofuran) were searched within the Guaymas metagenome. Notably, this chimney sample seemed to have the potential to degrade toluene, ethylbenzene and o-xylene (Table 5), yet no such evidence for benzene or the rest has been detected.

**Table 5:** **List of annotated gene features involved in degradation of hydrocarbons, absense of gene was marked with “–”**.

**Substrate**	**Enzyme (EC)**	**Best hit contig**	**Metagenome best hit organism**	**Similarity % (e-value)**
n-Alkanes	*assA1*	Contig00511	*Desulfoglaeba alkanexedens AK-01*	78 (5.2e-27)
	*assA2*	Contig45077	*Desulfoglaeba alkanexedens AK-01*	95 (9e-10)
Aromatic hydrocarbons	*bssA*	Contig28667	*Desulfotomaculum sp. Ox39*	76 (1.7e-6)
	*bssB*	–	–	–
	*bssC*	Contig46590	*Desulfatibacillum alkenivorans AK-01*	35 (2e-7)
	*bssD*	Contig29655	*Desulfatibacillum alkenivorans AK-01*	67 (1e-35)
	*bssE*	Contig46456	*Azoarcus sp.*	72 (1e-6)
	*bssG*	Contig46590	*Desulfatibacillum alkenivorans AK-01*	79 (1e-6)
Toluene	toluene 4-monooxygenase	Contig31764	*Dechloromonas aromatica (strain RCB)*	77 (1.6e-64)
Ethylbenzene	ethylbenzene dehydrogenase	Contig32722	*Desulfococcus oleovorans Hxd3*	86 (3.4e-145)
o-Xylene	o-xylene monooxygenase	Contig31764	*Dechloromonas aromatica (strain RCB)*	77 (1.6e-64)

### Comparative metagenomic analysis

**Figure 3:**
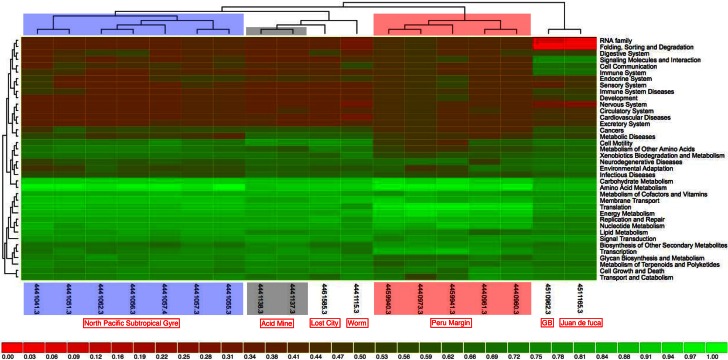
**The data was compared to KEGG database using a maximum e-value of 1e^−5^, a minimum identity of 30%.** The heatmap was clustered using ward clustering with Canberra distance metric and grouped at level 2 of KEGG annotation. The shading is proportional to the normalized values (0–1) calculated on the functional enrichment of each category per sample.

Clustering on (KEGG) functional gene categories was conducted among different environmental samples (Figure 3). Guaymas chimney sample 4558-6 (4510962.3) was clustered most closely with chimney sample from Juan de Fuca (4510965.3). Both of these two metagenomes were almost depleted in categories of RNA family and folding, sorting and degradation, while they showed higher abundance in the categories of signaling molecules, and interaction and cell communication. These features might be highly related to the specific environmental conditions where these two chimney samples were collected. The metagenome of the Lost City chimney sample (4461585.3) was not grouped with the above two chimney samples. The two biofilm samples from acid mine drainage (4441137.3 and 4441138.3) shared similar functional profiles in the heatmap, adjacent to the chimney sample from Lost City and the gutless worm sample (4441115.3). Samples from the North Pacific Subtropical Gyre microbial communities (4441051.3, 4441057.4, 4441055.3, 4441041.3, 4441057.3, 4441062.3, and 4441056.3) were grouped next to each other in the functional heatmap, and a similar pattern was observed for Peru Margin sediments samples (4440960.3, 4440961.3, 4440973.3, 4459940.3, and 4459941.3). Enzymes involved in hydrocarbon biodegradation in metagenomes from different environments were listed in Table A3. When compared to metagenomes from different environments, 4458-6 from Guaymas hydrothermal vent chimney was the only one with enzymes for biodegradation of toluene, ethylbenzene, and o-xylene (Table A3), highlighting its metabolic potential in degrading hydrocarbons in the native oil-immersed condition.

## Discussion

Advances in sequencing technologies have made microbial diversity studies easier and more accurate. However, biases were introduced during sequencing and analyzing of environmental sequences. For instance, biases were generated when multiple reads were produced for a unique DNA fragment in a random manner. Such biases might result in an inaccurate representation of the fragments and lead to misleading conclusions. Therefore, strict quality control of the sequenced reads should be done. In this study, a decent sequencing coverage has been reached (Figure 1) and we are in the position to investigate the taxonomic diversity as well as the metabolic potential of this Guaymas chimney sample.

### Diversity at different taxonomic levels

Estimated from both 16S rRNA sequences as well as taxonomic classifications based on blast hits, the proportion of bacteria in the community was about 60–75%, and 25–30% for archaea. Bacteria were dominated by Proteobacteria, while Euryarchaeota were most abundant among the archaea. So far, sulfate-reducing bacteria and archaea have been isolated in vent chimneys, with either high or low growth temperature (30–90°C) (Burggraf et al., 1990; Audiffrin et al., 2003; Moussard et al., 2004). Sulfate reduction may occur at temperatures up to 110°C in hot sediments from Guaymas hydrothermal field (Jørgensen et al., 1992). In this study, a high proportion (at least 21.2%) of sequences from this chimney metagenome was potentially originated from SRP (Table 3), a large and extremely diverse physiological group of anaerobic microorganisms. Sulfate-reducing bacteria and archaea were capable of degrading a wide range of organic substrates (Widdel and Bak, 1992; Widdel and Rabus, 2001), including petroleum-based products that were discussed in this study. The presence of large numbers of SRP in the oil-immersed chimney suggested that the microbial community had the potential of hydrocarbon biodegradation, which were likely to be coupled with sulfate reduction. Besides, most of the retrieved sequences were estimated to originate from thermophilc microorganisms such as *Archaeoglobus*, heterotrophic *Thermococcales* and *Thermodesulfobacteriaceae*, reflecting the influence of the high temperature on the structure of the microbial community.

### Metabolic potential for hydrocarbon degradation

Deep-sea environments have been characterized by the lack of easily biodegradable OM, thus genes related to the degradation of refractory OM have been extensively recovered from the metagenomes of deep oceans (Martin-Cuadrado et al., 2007). Moreover, bacteria isolated from the deep sea have been shown to be capable of degrading refractory OM such as chitin and cellulose (Hedges et al., 2000; Vezzi et al., 2005; Wang et al., 2008). Here, the potential of a chimney microbial community for refractory OM degradation was evaluated. Genes coding for chitin degradation were not found in the metagenome, while all the genes involved in the degradation of cellulose and fatty acids, as well as a few key enzymes in the breakdown of lipid and pullulan were identified, which probably reflected in part the input of terrestrial OM circulating in this Guaymas vent field. As most of the identified enzymes had a presumably thermophilic origin (Table 5 and Table A2), and thermophilic microorganisms were predominant in the chimney sample (Table 3), it was likely that these enzymes had some degree of heat tolerance. Additionally, microorganisms of this Guaymas chimney were predicted to have the potential to degrade toluene, ethylbenzene, and o-xylene. Notably, anaerobic hydrocarbon degrading microorganisms have been successfully enriched and most extensively studied with the benzene-toluene-ethylbenzene-xylenes (BTEX) group of petroleum hydrocarbons (Stockton et al., 2009). For example, pure culture strain EbS7 was isolated from the sediments of Guaymas Basin which was reported with the ethylbenzene-dependent sulfate reduction (Kniemeyer et al., 2003). This Guaymas chimney sample was oil immersed, thus it was not surprising to see the presence of genes involved in the degradations of petroleum hydrocarbons (Table 5). Our data further highlighted the potential of this microbial community using the fumarate addition pathway for the degradation of aromatic and aliphatic hydrocarbons. Identification of genes coding for hydrocarbon degradation would advance characterizations of the potential source of electrons and energy, as well as the roles of this chimney microbial community had played in its native environment. Notably, this Guaymas chimney metagenome seemed to be the only one (among all the samples included in the comparative metagenomic analysis) with metabolic potential for hydrocarbons biodegradation (Table A3). Enzymes activated by oxygen (namely active under aerobic conditions) were not identified in our metagenome, consistent with the strict anaerobic condition where this chimney sample was collected. Metagenomic analysis suggested that degradation of a variety of petroleum hydrocarbons by SRP might play an important role in the energy metabolism of this chimney microbial community.

### Comparison among different samples

For the moment, only three metagenomes from hydrothermal vent chimney are available, one from Juan de Fuca (Xie et al., 2011), one from Lost City (Brazelton and Baross, 2009) and the last one was our chimney sample 4458-6 from Guaymas Basin. The whole metagenome-based comparison (Figure 3) showed that Lost City was not clustered next to the other two chimney samples, which could be due to the fact that the Lost City sample was collected from a white carbonate chimney (CC) rather than a back sulfide chimney (SC) as the other two samples were. When considering the fact that the dominant energy sources for SC and CC were significant different (i.e., metal sulfides in SC and reduced volatiles such as hydrogen, methane in CC, respectively), the genomic differences between the two types of chimneys become clear as different energy metabolisms were promoted accordingly. Moreover, the venting fluid of Lost City had a pH of 9–11 and temperatures lower than 90°C, whereas that of Juan de Fuca sample was acidic and hot (temperature around 310°C); the vent fluid of our Guaymas chimney sample represented an intermediate temperature regime (190°C). Differences in environmental factors (such as temperature, pH, and physico-chemical gradients) between white and black chimneys could also have a significant influence on the distribution of functional gene categories, and as a result the three chimney samples were not clustered next to each other. On the other hand, when looking at specific gene categories, metagenomes from hydrothermal vent chimneys (Guaymas Basin, Juan de Fuca and Lost City) kept a larger collection of genes involved in recombination and repair, chemotaxis and flagellar assembly, as well as transposases. This could be a chimney-specific feature, reflecting adaptations of microorganisms and the microbial community to the extreme fluctuating chemical and physical conditions that characterize deep-sea hydrothermal vent chimneys.

### Conflict of interest statement

The authors declare that the research was conducted in the absence of any commercial or financial relationships that could be construed as a potential conflict of interest.

## Appendix

**Table A1:** **Taxonomic information based on assigned blastx results to each domain**.

**Domain**	**No. of hits assigned**	**% of hits assigned**
Bacteria	21792	58.3
Archaea	11249	30.1
Eukaryota	224	0.6
Virus	88	0.2
Unassigned	4019	10.8

**Table A2:** **List of annotated protein features involved in degradation processes**.

**Substrate: step enzyme**	***Best BLAST hit organism***
**STEP [CELLULOSE]: → CELLULOSE CELLOBIOSE**	
Endo-1,4-beta-glucanase	*Thermotoga neapolitana*^*^
Cellulase	*Thermosipho melanesiensis BI429*^*^
	*Methanococcoides burtonii DSM 6242*
Endoglucanase	*Archaeoglobus fulgidus DSM 4304*^*^
Cellobiose → beta-D-Glucose	
Beta-glucosidase	*Dictyoglomus thermophilum H-6-12 (2)*
**STEP [FATTY ACIDS]: FATTY ACIDS → HEXA-DECANOYL-COA**	
Long chain fatty acid CoA ligase	*Bacillus thuringiensis serovar huazhongensis BGSC 4BD1*
	*Bacteroides sp.2_1_7*
	*Bacillus cereus BDRD-ST196*
	*Desulfobacterium autotrophicum HRM2(2)*
	*Carboxydothermus hydrogenoformans Z-2901*^*^
	*Archaeoglobus fulgidus DSM 4304 (16)*^*^
Acyl-CoA synthetase	*Picrophilus torridus DSM 9790*^*^
	*Syntrophus aciditrophicus SB*
	*Desulfatibacillum alkenivorans AK-01*
	*Archaeoglobus fulgidus DSM 4304 (6)*^*^
**Hexa-decanoyl-CoA → Trans-hexadec-2-enoyl-CoA**	
Acyl-CoA dehydrogenase	*Pseudomonas aeruginosa PA7*
	*Marinobacter algicola DG893*
	*Colwellia psychrerythraea 34H*
	*Oceanobacter sp. RED65*
	*Oceanospirillum sp. MED92*
	*Gamma proteobacterium NOR51-B*
	*Brucella abortus bv. 1 str. 9-941*
	*Labrenzia alexandrii DFL-11*
	*Brucella suis bv. 5 str. 513*
	*Cytophaga hutchinsonii ATCC 33406*
	*Candidatus Cloacamonas acidaminovorans*
	*Bacillus clausii KSM-K16*
	*Archaeoglobus fulgidus DSM 4304 (16)*^*^
**Trans-hexadec-2-enoyl-CoA → (S)-3-Hydroxy-hexa-decanoyl-CoA**	
Beta-hydroxyacyl-CoA dehydrase	*Desulfuromonas acetoxidans DSM 684*
	*Desulfococcus oleovorans Hxd3 (4)*
Enoyl-CoA dydratase	*Burkholderia multivorans CGD2M*
	*Acidaminococcus sp. D21*
	*Thermosinus carboxydivorans Nor1*
	*Desulfococcus oleovorans Hxd3 (4)*
	*Desulfatibacillum alkenivorans AK-01*
	*Geobacter bemidjiensis Bem*
	*Candidatus Koribacter versatilis Ellin345*
	*Marinomonas sp. MWYL1*
	*Colwellia psychrerythraea 34H*
	*Carboxydothermus hydrogenoformans Z-2901*^*^
	*Archaeoglobus fulgidus DSM 4304*^*^
	*Rubrobacter xylanophilus DSM 9941 (2)*^*^
**(S)-3-Hydroxy-hexa-decanoyl-CoA → 3-Oxo-hexa-decanoyl-CoA**	
3-Hydroxyacyl-CoA dehydrogenase	*Gallionella ferruginea ES-2*
	*Desulfococcus oleovorans Hxd3*
	*Xanthobacter autotrophicus Py2*
	*Rhodospirillum centenum SW*
	*Geobacter uraniireducens Rf4*
	*Archaeoglobus fulgidus DSM 4304 (12)*^*^
Beta-hydroxyacyl dehydrogenase	*Desulfococcus oleovorans Hxd3 (4)*
	*Desulfuromonas acetoxidans DSM 684*
3-Ketoacyl-CoA thiolase	*Colwellia psychrerythraea 34H*
	*Halobacterium sp. NRC-1*
	*Anaeromyxobacter dehalogenans 2CP-C*
	*Archaeoglobus fulgidus DSM 4304 (6)*^*^
Thiolase	*Geobacter metallireducens GS-15*
	*Polaromonas sp. JS666*
**STEP [PULLULAN]: PULLULAN → PANOSE**	
Pullulanase	*Thermococcus onnurineus NA1(3)*^*^
	*Thermococcus hydrothermalis*^*^
	*Thermotoga sp. EMP*
Amylopullulanase	*Thermococcus sp. AM4*^*^
**STEP [LIPID]: LIPID → GLYCEROL**	
Glycerate kinase	*Thermoanaerobacter sp. X513*^*^
Aldehyde dehydrogenase	*Desulfitobacterium dichloroeliminans LMG P-21439*
	*Rhodothermus marinus SG0.5JP17-172*
	*Melioribacter roseus P3M*
	*Thermoplasma volcanium GSS1*^*^
	*Calditerrivibrio nitroreducens DSM 19672*
	*Uncultured Acidobacteria bacterium*
Glycerol dehydrogenase	*Archaeoglobus fulgidus DSM 4304*^*^

Thermalphilic species were marked with an asterisk.

**Table A3:** **List of enzymes involved in hydrocarbon biodegradation in metagenomes from different environments, presence was marked with “+,” while absence with “−”**.

	**Transposase**		**Toluene**	**Ethylbenzene**	**o-Xylene**
	**No. of hits**	**%**	**Presence**	**Presence**	**Presence**
4558-6	313	1.32	+	+	+
Juan de Fuca	158	1.10	−	−	−
Lost City	1387	9.64	−	−	−
Acid mine drainage	119	2.27	−	−	−
5way acid mine			−	−	−
Worm	200	2.80	−	−	−
Peru_ori1	36	0.49	−	−	−
Peru_amp1	10	0.18	−	−	−
Peru_amp16	6	0.10	−	−	−
Peru_amp32	12	0.11	−	−	−
Peru_amp50^*^	6	0.08	−	+	−
HOT_10	1	0.03	−	−	−
HOT_70	3	0.08	−	−	−
HOT_130	2	0.08	−	−	−
HOT_200	3	0.09	−	−	−
HOT_500	13	0.32	−	−	−
HOT_770	15	0.31	−	−	−
HOT_4000	49	1.03	−	−	−

* Hit property: e-value 4.0e-12, similarity 50%, ethylbenzene dehydrogenase alpha subunit.

## References

[B1] Altschul S. F. Madden T. L. Schaffer A. A. Zhang J. Zhang Z. Miller W. (1997). Gapped BLAST and PSI-BLAST: a new generation of protein database search programs. Nucleic Acids Res. 25, 3389–3402 10.1093/nar/25.17.3389 9254694 PMC146917

[B2] Amann R. I. Ludwig W. Schleifer K. H. (1995). Phylogenetic identification and *in situ* detection of individual microbial cells without cultivation. Microbiol. Rev. 59, 143–169 7535888 10.1128/mr.59.1.143-169.1995 PMC239358

[B3] Audiffrin C. Cayol J. L. Joulian C. Casalot L. Thomas P. Garcia J. L. (2003). *Desulfonauticus submarinus* gen. nov., sp. nov., a novel sulfate-reducing bacterium isolated from a deep-sea hydrothermal vent. Int. J. Syst. Evol. Microbiol. 53, 1585–1590 13130052 10.1099/ijs.0.02551-0

[B4] Bazylinski D. A. Farrington J. W. Jannasch H. W. (1988). Hydrocarbons in surface sediments from a Guaymas Basin hydrothermal vent site. Org. Geochem. 12, 547–559 10.1016/0146-6380(88)90146-5

[B5] Biddle J. F. Fitz-Gibbon S. Schuster S. C. Brenchley J. E. House C. H. (2008). Metagenomic signatures of the Peru Margin subseafloor biosphere show a genetically distinct environment. Proc. Natl. Acad. Sci. U.S.A. 105, 10583–10588 10.1073/pnas.0709942105 18650394 PMC2492506

[B6] Brazelton W. J. Baross J. A. (2009). Abundant transposases encoded by the metagenome of a hydrothermal chimney biofilm. ISME J. 3, 1420–1424 10.1038/ismej.2009.79 19571895

[B7] Brazelton W. J. Ludwig K. A. Sogin M. L. Andreishcheva E. N. Kelley D. S. Shen C. C. (2010). Archaea and bacteria with surprising microdiversity show shifts in dominance over 1,000-year time scales in hydrothermal chimneys. Proc. Natl. Acad. Sci. U.S.A. 107, 1612–1617 10.1073/pnas.0905369107 20080654 PMC2824366

[B8] Burggraf S. Jannasch H. W. Nicolaus B. Stetter K. O. (1990). *Archaeoglobus profundus* sp. nov., represents a new species within the sulfate-reducing Archaebacteria. Syst. Appl. Microbiol. 13, 24–28 10.1016/S0723-2020(11)80176-1

[B9] Callaghan A. V. Davidova I. A. Savage-Ashlock K. Parisi V. A. Gieg L. M. Suflita J. M. (2010). Diversity of benzyl- and alkylsuccinate synthase genes in hydrocarbon-impacted environments and enrichment cultures. Environ. Sci. Technol. 44, 7287–7294 10.1021/es1002023 20504044

[B10] Delong E. F. Preston C. M. Mincer T. Rich V. Hallam S. J. Frigaard N. U. (2006). Community genomics among stratified microbial assemblages in the ocean's interior. Science 311, 496–503 10.1126/science.1120250 16439655

[B11] Dick G. J. Tebo B. M. (2010). Microbial diversity and biogeochemistry of the Guaymas Basin deep-sea hydrothermal plume. Environ. Microbiol. 12, 1334–1347 10.1111/j.1462-2920.2010.02177.x 20192971

[B12] Distel D. L. Lane D. J. Olsen G. J. Giovannoni S. J. Pace B. Pace N. R. (1988). Sulfur-oxidizing bacterial endosymbionts: analysis of phylogeny and specificity by 16S rRNA sequences. J. Bacteriol. 170, 2506–2510 3286609 10.1128/jb.170.6.2506-2510.1988 PMC211163

[B13] Elsgaard L. Isaksen M. F. Jørgensen B. B. Alayse A. M. Jannasch H. W. (1994). Microbial sulfate reduction in deep-sea sediments at the Guaymas Basin hydrothermal vent area: influence of temperature and substrates. Geochim. Cosmochim. Acta 58, 3335–3343 10.1016/0016-7037(94)90089-2

[B14] Flores G. E. Campbell J. H. Kirshtein J. D. Meneghin J. Podar M. Steinberg J. I. (2011). Microbial community structure of hydrothermal deposits from geochemically different vent fields along the Mid-Atlantic Ridge. Environ. Microbiol. 13, 2158–2171 10.1111/j.1462-2920.2011.02463.x 21418499

[B15] Galushko A. Minz D. Schink B. Widdel F. (1999). Anaerobic degradation of naphthalene by a pure culture of a novel type of marine sulphate-reducing bacterium. Environ. Microbiol. 1, 415–420 10.1046/j.1462-2920.1999.00051.x 11207761

[B16] Gao J. Ellis L. B. Wackett L. P. (2010). The University of Minnesota Biocatalysis/Biodegradation Database: improving public access. Nucleic Acids Res. 38, D488–D491 10.1093/nar/gkp771 19767608 PMC2808978

[B17] Gomez-Alvarez V. Teal T. K. Schmidt T. M. (2009). Systematic artifacts in metagenomes from complex microbial communities. ISME J. 3, 1314–1317 10.1038/ismej.2009.72 19587772

[B18] He Z. Deng Y. Van Nostrand J. D. Tu Q. Xu M. Hemme C. L. (2010). GeoChip 3.0 as a high-throughput tool for analyzing microbial community composition, structure and functional activity. ISME J. 4, 1167–1179 10.1038/ismej.2010.46 20428223

[B19] Hedges J. I. Eglinton G. Hatcher P. G. Kirchman D. L. Arnosti C. Derenne S. (2000). The molecularly-uncharacterized component of nonliving organic matter in natural environments. Org. Geochem. 31, 945–958 10.1016/S0146-6380(00)00096-6

[B20] Huber J. A. Cantin H. V. Huse S. M. Welch D. B. Sogin M. L. Butterfield D. A. (2010). Isolated communities of Epsilonproteobacteria in hydrothermal vent fluids of the Mariana Arc seamounts. FEMS Microbiol. Ecol. 73, 538–549 10.1111/j.1574-6941.2010.00910.x 20533947

[B21] Huber J. A. Mark Welch D. B. Morrison H. G. Huse S. M. Neal P. R. Butterfield D. A. (2007). Microbial population structures in the deep marine biosphere. Science 318, 97–100 10.1126/science.1146689 17916733

[B22] Huson D. H. Auch A. F. Qi J. Schuster S. C. (2007). MEGAN analysis of metagenomic data. Genome Res. 17, 377–386 10.1101/gr.5969107 17255551 PMC1800929

[B23] Jannasch H. W. Mottl M. J. (1985). Geomicrobiology of deep-sea hydrothermal vents. Science 229, 717–725 10.1126/science.229.4715.717 17841485

[B24] Jørgensen B. B. Isaksen M. F. Jannasch H. W. (1992). Bacterial sulfate reduction above 100{degrees}C in deep-sea hydrothermal vent sediments. Science 258, 1756–1757 10.1126/science.258.5089.1756 17831655

[B25] Jørgensen B. B. Zawacki L. X. Jannasch H. W. (1990). Thermophilic bacterial sulfate reduction in deep-sea sediments at the Guaymas Basin hydrothermal vents (Gulf of California). Deep-Sea Res. I 37, 695–710 10.1016/0198-0149(90)90099-H

[B26] Kallmeyer J. Boetius A. (2004). Effects of temperature and pressure on sulfate reduction and anaerobic oxidation of methane in hydrothermal sediments of Guaymas Basin. Appl. Environ. Microbiol. 70, 1231–1233 10.1128/AEM.70.2.1231-1233.2004 14766611 PMC348843

[B27] Kawka O. E. Simoneit B. R. T. (1994). Hydrothermal pyrolysis of organic matter in Guaymas Basin. I. Comparison of hydrocarbon distributions in subsurface sediments and seabed petroleums. Org. Geochem. 22, 947–978 10.1016/0146-6380(94)90031-0

[B29] Kleindienst S. Ramette A. Amann R. Knittel K. (2012). Distribution and in situ abundance of sulfate-reducing bacteria in diverse marine hydrocarbon seep sediments. Environ. Microbiol. 14, 2689–2710 10.1111/j.1462-2920.2012.02832.x 22882476

[B30] Kniemeyer O. Fischer T. Wilkes H. Glockner F. O. Widdel F. (2003). Anaerobic degradation of ethylbenzene by a new type of marine sulfate-reducing bacterium. Appl. Environ. Microbiol. 69, 760–768 10.1128/AEM.69.2.760-768.2003 12570993 PMC143655

[B32] Lam P. Cowen J. P. Jones R. D. (2004). Autotrophic ammonia oxidation in a deep-sea hydrothermal plume. FEMS Microbiol. Ecol. 47, 191–206 10.1016/S0168-6496(03)00256-3 19712334

[B33] Martens C. S. (1990). Generation of short chain organic acid anions in hydrothermally altered sediments of the Guaymas Basin, Gulf of California. Appl. Geochem. 5, 71–76 10.1016/0883-2927(90)90037-6

[B34] Martin-Cuadrado A. B. Lopez-Garcia P. Alba J. C. Moreira D. Monticelli L. Strittmatter A. (2007). Metagenomics of the deep Mediterranean, a warm bathypelagic habitat. PLoS ONE 2:e914 10.1371/journal.pone.0000914 17878949 PMC1976395

[B35] Meyer F. Paarmann D. D'Souza M. Olson R. Glass E. M. Kubal M. (2008). The metagenomics RAST server - a public resource for the automatic phylogenetic and functional analysis of metagenomes. BMC Bioinformatics 9:386 10.1186/1471-2105-9-386 18803844 PMC2563014

[B36] Moussard H. L'Haridon S. Tindall B. J. Banta A. Schumann P. Stackebrandt E. (2004). *Thermodesulfatator indicus* gen. nov., sp. nov., a novel thermophilic chemolithoautotrophic sulfate-reducing bacterium isolated from the Central Indian Ridge. Int. J. Syst. Evol. Microbiol. 54, 227–233 10.1099/ijs.0.02669-0 14742485

[B37] Musat F. Widdel F. (2008). Anaerobic degradation of benzene by a marine sulfate-reducing enrichment culture, and cell hybridization of the dominant phylotype. Environ. Microbiol. 10, 10–19 10.1111/j.1462-2920.2007.01425.x 18211263

[B38] Niu B. Fu L. Sun S. Li W. (2010). Artificial and natural duplicates in pyrosequencing reads of metagenomic data. BMC Bioinformatics 11:187 10.1186/1471-2105-11-187 20388221 PMC2874554

[B39] Ogata H. Goto S. Sato K. Fujibuchi W. Bono H. Kanehisa M. (1999). KEGG: kyoto encyclopedia of genes and genomes. Nucleic Acids Res. 27, 29–34 10.1093/nar/27.1.29 9847135 PMC148090

[B40] Petersen J. M. Zielinski F. U. Pape T. Seifert R. Moraru C. Amann R. (2011). Hydrogen is an energy source for hydrothermal vent symbioses. Nature 476, 176–180 10.1038/nature10325 21833083

[B41] Powell S. Szklarczyk D. Trachana K. Roth A. Kuhn M. Muller J. (2012). eggNOG v3.0: orthologous groups covering 1133 organisms at 41 different taxonomic ranges. Nucleic Acids Res. 40, D284–D289 10.1093/nar/gkr1060 22096231 PMC3245133

[B42] Reysenbach A. L. Shock E. (2002). Merging genomes with geochemistry in hydrothermal ecosystems. Science 296, 1077–1082 10.1126/science.1072483 12004120

[B43] Rho M. Tang H. Ye Y. (2010). FragGeneScan: predicting genes in short and error-prone reads. Nucleic Acids Res. 38:e191 10.1093/nar/gkq747 20805240 PMC2978382

[B44] Roussel E. G. Konn C. Charlou J. L. Donval J. P. Fouquet Y. Querellou J. (2011). Comparison of microbial communities associated with three Atlantic ultramafic hydrothermal systems. FEMS Microbiol. Ecol. 77, 647–665 10.1111/j.1574-6941.2011.01161.x 21707671

[B45] Rüter P. Rabus R. Wilkes H. Aeckersberg F. Rainey F. A. Jannasch H. W. (1994). Anaerobic oxidation of hydrocarbons in crude oil by new types of sulphate-reducing bacteria. Nature 372, 455–458 10.1038/372455a0 7984238

[B46] Stockton A. M. Chiesl T. N. Scherer J. R. Mathies R. A. (2009). Polycyclic aromatic hydrocarbon analysis with the Mars organic analyzer microchip capillary electrophoresis system. Anal. Chem. 81, 790–796 10.1021/ac802033u 19072718

[B47] Tatusov R. L. Fedorova N. D. Jackson J. D. Jacobs A. R. Kiryutin B. Koonin E. V. (2003). The COG database: an updated version includes eukaryotes. BMC Bioinformatics 4:41 10.1186/1471-2105-4-41 12969510 PMC222959

[B48] Teske A. Hinrichs K. U. Edgcomb V. De Vera Gomez A. Kysela D. Sylva S. P. (2002). Microbial diversity of hydrothermal sediments in the Guaymas Basin: evidence for anaerobic methanotrophic communities. Appl. Environ. Microbiol. 68, 1994–2007 11916723 10.1128/AEM.68.4.1994-2007.2002 PMC123873

[B49] Tyson G. W. Chapman J. Hugenholtz P. Allen E. E. Ram R. J. Richardson P. M. (2004). Community structure and metabolism through reconstruction of microbial genomes from the environment. Nature 428, 37–43 10.1038/nature02340 14961025

[B50] Vezzi A. Campanaro S. D'Angelo M. Simonato F. Vitulo N. Lauro F. M. (2005). Life at depth: photobacterium profundum genome sequence and expression analysis. Science 307, 1459–1461 10.1126/science.1103341 15746425

[B51] Von Damm K. L. (1990). Seafloor hydrothermal activity: black smoker chemistry and chimneys. Annu. Rev. Earth Planet Sci. 18, 173–204 10.1146/annurev.ea.18.050190.001133

[B52] Von Damm K. L. Edmond J. M. Measures C. I. Grant B. (1985). Chemistry of submarine hydrothermal solutions at Guaymas Basin, Gulf of California Geochimica et Cosmochimica Acta 49, 2221–2237 10.1016/0016-7037(85)90223-6

[B53] Wang F. Wang J. Jian H. Zhang B. Li S. Wang F. (2008). Environmental adaptation: genomic analysis of the piezotolerant and psychrotolerant deep-sea iron reducing bacterium Shewanella piezotolerans WP3. PLoS ONE 3:e1937 10.1371/journal.pone.0001937 18398463 PMC2276687

[B54] Wang F. Zhou H. Meng J. Peng X. Jiang L. Sun P. (2009). GeoChip-based analysis of metabolic diversity of microbial communities at the Juan de Fuca Ridge hydrothermal vent. Proc. Natl. Acad. Sci. U.S.A. 106, 4840–4845 10.1073/pnas.0810418106 19273854 PMC2660763

[B55] Weber A. Jørgensen B. B. (2002). Bacterial sulfate reduction in hydrothermal sediments of the Guaymas Basin, Gulf of California, Mexico. Deep Sea Res. I 149, 827–841

[B56] Welhan J. A. (1988). Origins of methane in hydrothermal systems. Chem. Geol. 71, 183–198 11539452

[B57] Widdel F. Bak F. (1992). Gram-negative mesophilic sulfate-reducing bacteria, in The Prokaryotes, eds Balows A. Trüper H. G. Dworkin M. Harder W. Schleifer K. H. (New York, NY: Springer-Verlag), 3352–3378

[B58] Widdel F. Rabus R. (2001). Anaerobic biodegradation of saturated and aromatic hydrocarbons. Curr. Opin. Biotechnol. 12, 259–276 11404104 10.1016/s0958-1669(00)00209-3

[B59] Woyke T. Teeling H. Ivanova N. N. Huntemann M. Richter M. Gloeckner F. O. (2006). Symbiosis insights through metagenomic analysis of a microbial consortium. Nature 443, 950–955 10.1038/nature05192 16980956

[B60] Wu S. Zhu Z. Fu L. Niu B. Li W. (2011). WebMGA: a customizable web server for fast metagenomic sequence analysis. BMC Genomics 12:444 10.1186/1471-2164-12-444 21899761 PMC3180703

[B61] Xie W. Wang F. Guo L. Chen Z. Sievert S. M. Meng J. (2011). Comparative metagenomics of microbial communities inhabiting deep-sea hydrothermal vent chimneys with contrasting chemistries. ISME J. 5, 414–426 10.1038/ismej.2010.144 20927138 PMC3105715

[B62] Zerbino D. R. Birney E. (2008). Velvet: algorithms for de novo short read assembly using de Bruijn graphs. Genome Res. 18, 821–829 10.1101/gr.074492.107 18349386 PMC2336801

